# Surgical Practice in the Current COVID-19 Pandemic: A Rapid Systematic Review

**DOI:** 10.6061/clinics/2020/e1923

**Published:** 2020-05-11

**Authors:** Flávio Carneiro Hojaij, Lucas Albuquerque Chinelatto, Gustavo Henrique Pereira Boog, Júlia Adriana Kasmirski, João Vitor Ziroldo Lopes, Fernando Mauad Sacramento

**Affiliations:** I Departamento de Cirurgia, Laboratorio de Investigacao Medica (LIM 02), Faculdade de Medicina FMUSP, Universidade de Sao Paulo, Sao Paulo, SP, BR; II Faculdade de Medicina FMUSP, Universidade de Sao Paulo, Sao Paulo, SP, BR

**Keywords:** Surgery, Operation, SARS-CoV-2, COVID-19, Coronavirus, Recommendations

## Abstract

The coronavirus disease (COVID-19) outbreak started in Wuhan, China, in December 2019, and evolved into a global problem in a short period. The pandemic has led to many social and health-care challenges. In this context, surgery is an area that is facing the need for many adaptations. In this systematic literature review, we analyzed different perspectives concerning this situation, aiming to provide recommendations that could guide surgeons and entities toward screening, elective and emergency surgeries, decision making, and operating room management. A computerized search in PubMed, Scopus, and Scientific Electronic Library Online (SciELO) for relevant literature up to April 4, 2020, was performed. Articles were included if they were related to surgery dynamics in the context of the COVID-19 pandemic. Of the 281 articles found in our initial search and 15 articles from alternative sources, 39 were included in our review after a systematic evaluation. Concerning preoperative testing for severe acute respiratory syndrome coronavirus 2 infection, 29 (74.4%) articles recommended some kind of screening. Another major suggestion was postponing all (or at least selected) elective operations (29 articles, 74.4%). Several additional recommendations with respect to surgical practice or surgical staff were also assessed and discussed, such as performing laparoscopic surgeries and avoiding the use of electrocauterization. On the basis of the current literature, we concluded that any surgery that can be delayed should be postponed. COVID-19 screening is strongly recommended for all surgical cases. Moreover, surgical staff should be reduced to the essential members and provided with institutional psychological support.

## INTRODUCTION

On December 31, 2019, a cluster of patients with pneumonia of unknown cause was reported in China 1. These cases were epidemiologically linked to a seafood and wet animal wholesale market in Wuhan, Hubei Province. Further analysis through unbiased sequencing and isolation of airway epithelial cells revealed a previously unknown betacoronavirus, named 2019-nCOV (2019 novel coronavirus), which became the seventh member of the family of coronaviruses that infect humans 2. Early reports suggested the onset of a potential coronavirus outbreak because of the high reproduction number. By the end of January 2020, 7,734 cases were confirmed in China, with 90 others being reported in other countries including Thailand, Vietnam, Malaysia, Nepal, Sri Lanka, Japan, Republic of Korea, United States, India, Australia, Canada, France, and Germany. At this time, the World Health Organization (WHO) declared a Public Health Emergency of International Concern. On April 4, 2020, the most recent date covered by this review, the WHO reported 1,051,697 confirmed cases and 56,986 deaths in 207 countries affected by the epidemic 3, after coronavirus disease (COVID-19, named by the WHO on February 11, 2020) was declared a global pandemic on March 11, 2020 4.

COVID-19 was revealed to be a complex disease. Its replication rate (R0) of 2.0–3.0, death rate of approximately 5%, and the absence of immunological memory in humans, together with the time of adaptation for disease screening and notification, led to a rapid growth in the numbers of infected individuals worldwide 4,5. This phenomenon brought the attention to the greatest challenge posed by this disease: the high demand for health-care services *versus* the capability of health-care systems 5. The rapid growth in the numbers of infected individuals has drastically increased the demand for health services, which has led to social and health-care adaptations, ranging from social distancing to the reformulation of health-care delivery 6.

In this context, surgery is one of the areas facing the need for many adaptations. In many places, elective surgeries are preferably postponed and urgent/emergency surgeries – such as surgery for trauma and complicated hernias – are continued with some alterations (*e.g.*, reducing the number of surgical staff and modifying the organization of the operating room) 7. Many societies and authors have published different guidelines with respect to surgical protocols in this pandemic situation. In this review, we aimed to analyze different perspectives concerning this situation, with the objective of providing recommendations that could guide surgeons and entities toward screening, elective and emergency surgeries, decision making, and operating room management.

## MATERIAL AND METHODS

We performed a systematic literature review based on an online search in the PubMed (from the National Center for Biotechnology Information), Scientific Electronic Library Online (SciELO), and Scopus databases. The following terms were used in the search engine to find matching articles: (OPERATION OR SURGERY) AND (SARS-COV-2 OR COVID-19 OR CORONAVIRUS). Our initial search yielded 281 articles. Other articles sent to the authors by societies or colleagues were also included in the sample (n=15). Duplicates were initially deleted using the Endnote (Clarivate Analytics) reference engine. Articles that were not in English (Portuguese, Spanish, or German) were translated using online translation service. All article titles and abstracts were read by at least one author, and selected if relevant. For this review, an article was considered relevant if it (a) is related to the COVID-19 pandemic, (b) suggested approaches directly affecting surgery dynamics (*e.g.*, reducing staff numbers, remodeling the circulation of staff and patients in the operating room, using different protection methods for patients and staff, and adapting different equipment usage), (c) suggested any kind of screening for severe acute respiratory syndrome coronavirus 2 (SARS-CoV-2) for surgical patients, or (d) had any kind of recommendation about elective procedures. Papers focused on anesthetics and general hospital management were excluded.

For the articles selected for full-text reading, at least two authors read the contents. If those authors disagreed about including an article in our review, a third author read the paper to arrive at a consensus.

Data analysis was performed using the Google Sheets application (Google Inc.).

## RESULTS

### Records

**Figure 1:**
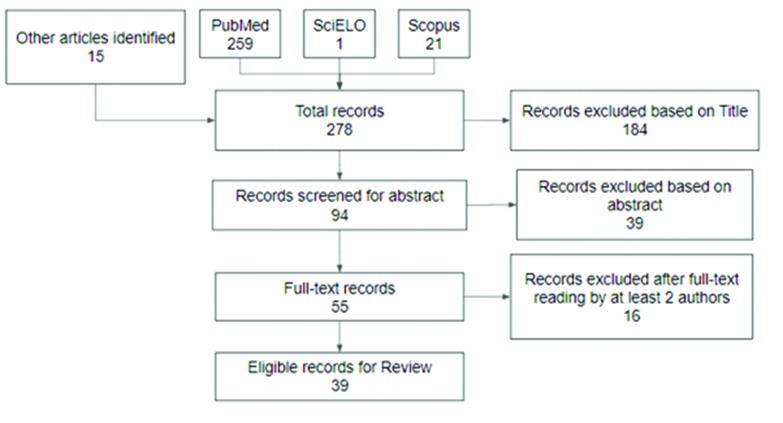
Selection diagram.

**Figure 2:**
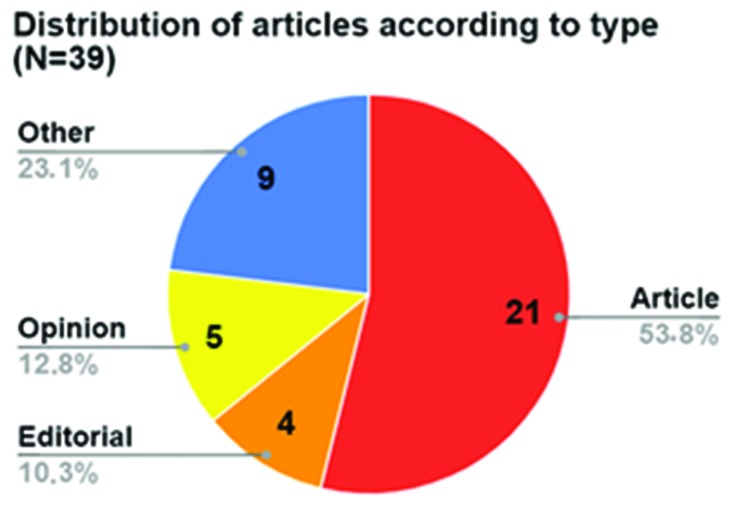
Distribution of articles according to type. The “Other” category included reviews, correspondences, and case reports.

Of the 281 articles found in our initial search and 15 articles from alternative sources, 39 were included in our review after a systematic evaluation (Figure 1). Of the 39 selected publications, 21 were research articles, 5 were opinion papers, 4 were editorials, and 9 were grouped as “others” (Figure 2).

**Figure 3:**
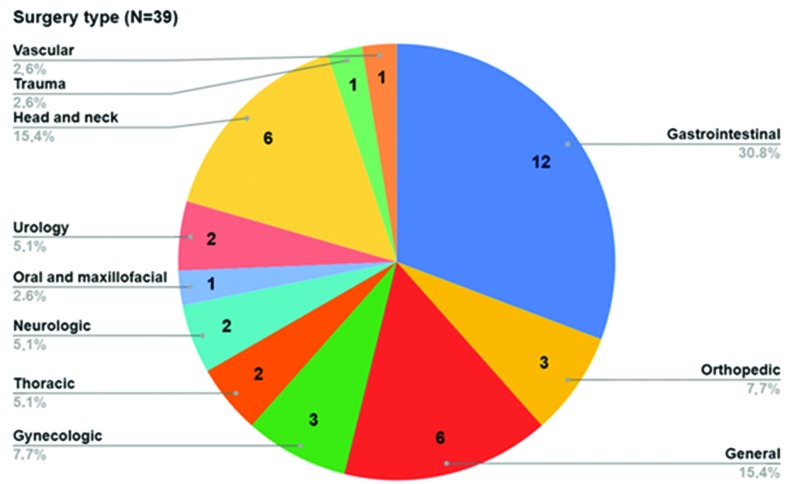
Distribution of articles according to the reported surgery type. Head and neck surgeries included otorhinolaryngology surgeries. Gastrointestinal surgeries included endoscopies.

Most of the articles were related to surgical interventions in the gastrointestinal tract, followed by articles on head and neck and general surgeries. Other, less frequent, surgeries were vascular, trauma, oral and maxillofacial and thoracic (Figure 3).

### COVID-19 Screening

**Figure 4:**
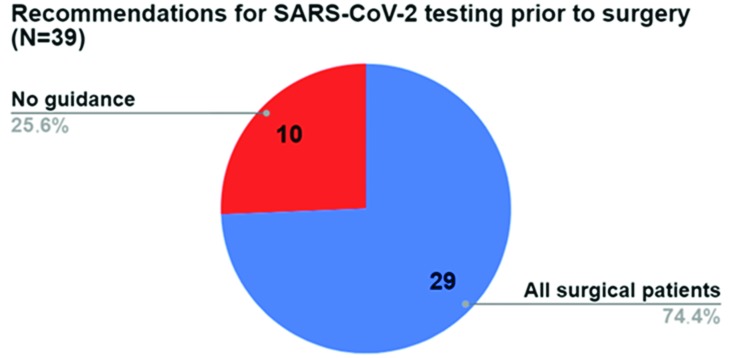
Distribution of articles according to recommendation for severe acute respiratory syndrome coronavirus 2 (SARS-CoV-2) screening. A total of 29 articles suggested some kind of screening, ranging from clinical evaluation to nasal swab testing, whereas 10 articles did not offer guidance. None of the articles negated the need for screening.

**Figure 5:**
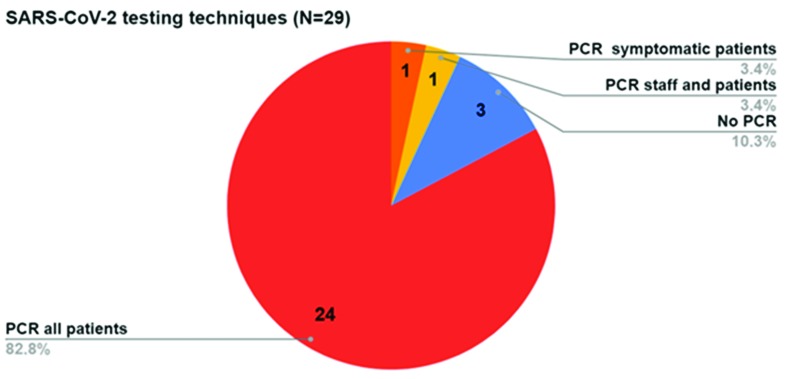
Distribution of 29 articles according to specific recommendations for severe acute respiratory syndrome coronavirus 2 (SARS-CoV-2) screening. “PCR symptomatic patients” (n=1) refers to the recommendation of testing only surgical patients with coronavirus disease symptoms. “PCR staff and patients” (n=1) refers to the recommendation of testing all surgical patients and surgical staff. “No PCR” (n=3) refers to the recommendation of using other methods of screening based on computed tomography scan, clinical examination, body temperature measurement, and blood tests. “**PCR all patients” (n=24) refers to the recommendation of testing all surgical patients through nasal swab testing or other kinds of PCR testing.** PCR: polymerase chain reaction

Twenty-nine articles recommended screening for SARS-CoV-2 in patients elected for surgery (Figure 4). The most prevalent recommendation was to perform polymerase chain reaction (PCR) testing of nasal swabs in all surgical patients. Other suggestions included screening through computed tomography, clinical examination, temperature measurement, and measurement of immune cells in blood samples, as well as testing only symptomatic patients (Figure 5).

### Surgical Recommendations

**Figure 6:**
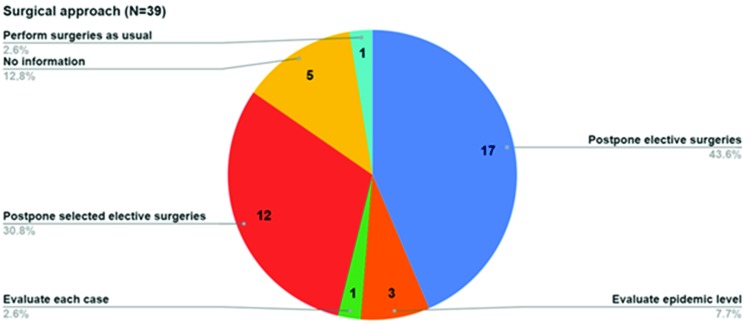
Distribution of articles according to recommendations related to delaying or continuing elective surgeries. “Evaluate epidemic level” refers to the recommendation of postponing elective surgeries according to epidemic stages. **All articles recommended taking precautions about infections in the surgical environment.**

With respect to surgical schedules, a little fewer than one-half of the articles recommended postponing all elective surgeries (Figure 6). Another prevalent suggestion was to select, from the group of elective surgeries, those that could be postponed. Around 13% of the articles did not offer any direct recommendations with respect to elective surgeries.

The articles also offered recommendations about surgical practice, including reorganization of the operating room schedule to provide only essential surgical services, guaranteeing psychological support to surgical staff, increasing protective measures to level 3 standards, and utilizing telemedicine for consultations that do not require physical evaluation. Table 1 summarizes the 10 most frequent and relevant recommendations and the article(s) that suggested them. (Note: Not all recommendations are included in the table.)

## DISCUSSION

The coronavirus pandemic is an unprecedented scenario for the field of surgery. Surgery for high-risk cases cannot be postponed; however, the SARS-CoV-2 virus has a high transmissibility and techniques that could allow surgeons to perform safer surgeries in the operating room are warranted.

Isolation is impossible to practice in the operating room because an anesthetist has to stand close to the patient to adequately perform intubation 40, which increases the exposure to aerosols that may be contaminated by the virus. Further, the virus also spreads through biological fluids, thus compromising the safety of the surgical environment in most cases.

Methods used to reduce contact have been suggested. Laparoscopies have been considered as a good way to diminish risks, because, in theory, a closed abdomen, which prevents the spread of gas or liquid, would decrease the possibility of contamination 36. Nonetheless, incidents during the exit of the *trocar* were reported, in which leakage of gas occurred in many cases, suggesting that laparotomy with extensive fluid drainage would be safer 26. Most hospitals agree that isolating the operating area is a better approach; however, this is difficult to accomplish. Operating inside closed doors can help reduce the number of infections among surgical staff members 15.

An article also suggested that SARS-CoV-2 could penetrate traditional surgical masks and gowns, thus necessitating the use of N95 masks and level 3 protection suits 23. Another significant concern is the procedure time, as greater exposure in the operating room leads to a higher risk of infection. Therefore, an article suggested that a team comprising the minimum number of experienced professionals should perform the surgery to optimize the procedure and reduce the surgery time 11. Another recommended approach for enhancing safety is selecting only known and reliable methods to reduce the risk of postoperative complications and prolonged hospitalization.

Trainee involvement must be reevaluated, as it can lead to an increase in procedure time and, consequently, the risk of exposure 12. The involvement of medical students in the clerkship and subinternship periods in teaching hospitals also leads to a similar problem as trainee involvement. The use of electrocautery is controversial because it produces a high concentration of contaminated gases 20. Therefore, an article suggested avoiding its use in a pandemic setting 28. Another article suggested allowing the use of electrocautery with some modifications, such as adjusting to the lowest possible effective power and employing a smoke absorption device 15.

Our review also found that it is relevant to strengthen the knowledge of health-care workers about hygiene measures during their shift. The constant stress caused by imminent contamination is known to make professionals more likely to experience mental health issues 20. Therefore, we advise hospitals to offer psychological support to their staff.

Most centers advise doctors to postpone elective surgeries for two main reasons: the risk of contamination is too high in the current epidemiological situation and medical equipment should be reserved for managing COVID-19 emergency cases 26. Online consultations are strongly recommended instead of hospital or clinic visits 27.

## CONCLUSION

Our main conclusion from this review is that any elective surgery that can be delayed should be postponed, taking into consideration that it may take 2–3 months for the health-care situation to return to normal. We also conclude that all surgical patients should be screened for COVID-19, with preference given to PCR tests. Regarding surgical practice recommendations, we believe that surgical staff should be reduced to the minimum, without compromising the procedure and any operation should be performed by the most experienced surgeon, so that the procedure time is reduced. An institutional workflow to assist medical staff in decision making and in dealing with mental health issues should also be established.

## AUTHOR CONTRIBUTIONS

Hojaij FC contributed to the conception and design of the study and provided advice to all other authors. Chinelatto LA, Boog GHP, Lopes JVZ, Kasmirski JA and Sacramento FM performed the literature search and wrote the manuscript. Hojaij FC reviewed the manuscript. All authors read and approved the final version of the manuscript.

## Figures and Tables

**Table 1:** General recommendations for surgical practice.

Recommendation	Number of articles	References
Reduce the circulation of health-care workers	23	8-30
Increase the personal protective equipment (PPE) indications	23	8, 11, 12, 15-24, 26, 28-36
Designate specific spaces for invasive/surgical treatments for all patients with COVID-19	17	8, 11, 12, 15-18, 20, 22-24, 28, 29, 31, 32, 37, 38
Operate on suspected or confirmed cases in negative-pressure operating rooms	15	11, 15-19, 26, 27, 32-34, 36, 38-40
Adopt level 3 protective measures	15	12, 15, 16-18, 21, 22, 24, 28, 32, 35, 36, 38, 40, 41
Develop a plan for providing essential operations	14	8, 13-16, 18-21, 27, 29, 32, 33, 42
Educate surgical staff about PPE and/or basic hygiene principles	13	8, 11-13, 15-18, 20, 25, 26, 29, 31
Adhere to online consultations/telemedicine	13	10, 13, 14, 20, 23-28, 34, 35, 39
Optimize the surgery time	12	8, 11, 13, 17, 22-25, 27, 35, 36, 41, 42
Offer psychological support to surgical staff	3	10, 15, 20
